# Ecological and evolutionary responses of earthworm holobionts to environmental changes

**DOI:** 10.1093/ismejo/wraf044

**Published:** 2025-03-09

**Authors:** Michael Opoku Adomako, Jing Wu, Fei-Hai Yu

**Affiliations:** School of Life and Environmental Sciences, Shaoxing University, Shaoxing 312000, Zhejiang, China; Institute of Wetland Ecology & Clone Ecology/Zhejiang Provincial Key Laboratory of Plant Evolutionary Ecology and Conservation, Taizhou University, Taizhou 318000, Zhejiang, China; Institute of Wetland Ecology & Clone Ecology/Zhejiang Provincial Key Laboratory of Plant Evolutionary Ecology and Conservation, Taizhou University, Taizhou 318000, Zhejiang, China; School of Life and Environmental Sciences, Shaoxing University, Shaoxing 312000, Zhejiang, China; Institute of Wetland Ecology & Clone Ecology/Zhejiang Provincial Key Laboratory of Plant Evolutionary Ecology and Conservation, Taizhou University, Taizhou 318000, Zhejiang, China

**Keywords:** animal–microbiota interactions, earthworm ecological groups, earthworm holobionts, eco-evolutionary processes, global change factors, gut microbial communities

## Abstract

Global environmental change substantially affects soil detritivores, including earthworms, impacting host–microbiota interactions and altering key soil biogeochemical processes such as litter decomposition. As microbial communities are inherently capable of rapid evolution, responses of earthworms and associated microbiota (i.e. earthworm holobionts) to global environmental change may likely involve the interplay of ecological and evolutionary processes and feedback. Although species-level responses of earthworms to global environmental change are well studied, the potential ecological and evolutionary responses of earthworm holobionts to environmental change remain unexplored. Here, we provide a conceptual framework to elaborate on the complex network of earthworm host–microbiota interactions that modify their traits in response to global environmental change, jointly shaping their ecology and evolution. Based on the literature, we synthesize evidence of global environmental change impacts on earthworm host–microbiota and discuss evidence of their ecological and evolutionary responses to environmental change. Lastly, we highlight the agro- and eco-system-level consequences of environmental change-mediated shift in earthworm host–microbiota functions. Soil legacies of environmental change have cascading detrimental impacts on the abundance, diversity, and functional dynamics of earthworm host–microbiota interactions in agriculture and ecosystems. The primary mechanisms driving such responses of earthworm hosts and associated microbial communities to environmental change include altered litter quality and host dietary preferences, competitive interactions and exclusion, habitat homogenization, and a shift in soil physicochemical and biological processes. Therefore, advancing knowledge of the intricate animal–microorganism interactions is crucial for belowground biodiversity management in a changing global environment.

## Introduction

Global environmental change (GEC) is impacting earthworm hosts and their associated microbiota, i.e. the earthworm holobiont [1–4], critically affecting vital biogeochemical processes, soil health, and ecosystem functioning [5–9]. As ecosystem engineers, earthworms improve soil structure and enhance organic matter mineralization and nutrient availability [10–12], contributing ~6.5% and 2.3% of global annual grain and legume crop production, respectively [13]. However, the global environment is changing fast due to increasing anthropogenic activities, substantially altering the cooperative roles of earthworms and their associated microbial communities [14]. Since microbial communities are inherently capable of rapid evolution [15, 16], responses of earthworm holobionts to GEC may likely involve the interplay of ecological and evolutionary processes and feedbacks. Although the species-level responses of earthworms and associated microbiota to GEC impacts have been extensively studied [17–19], the potential ecological and evolutionary responses of earthworm holobionts to GEC remain unexplored.

The earthworm gut harbors a diversity of microbial communities with distinct functional roles that promote soil health [11, 20, 21]. These gut microbiota are recruited based on the earthworm’s functional group, species, and habitat [22]. Earthworm–microbiota interactions provide mutual physiological, behavioral, and ecological support for each other’s growth, survival, and adaptation to environmental perturbation [23–26]. For instance, gut microbial communities of *Eisenia nordenskioldi* Eisen and *Drawida ghilarovi* Gates play integral roles in earthworm temperature adaptability and cellulose digestion [24]. Given the sensitivity of earthworms and their gut-associated microbial communities to changes in environmental conditions, a consistent trait shift in response to environmental stress could likely be developed into an adaptive strategy [27]. Increasing microorganism adaptation reduces the diversity of microbial communities, which has strong implications for their multifunctionality [28, 29]. Therefore, the ecological and evolutionary responses of earthworm holobionts to GEC are crucial for highlighting how animal host–microbiota interactions may jointly shape the ecology and evolution of each partner.

As a meta-organism, an earthworm holobiont represents a complex interdependent system, underscoring the need to consider the GEC responses of earthworm holobionts as a whole. In earthworm holobiont systems, however, the host predominantly initiates ecological responses to GEC that subsequently trigger a response in its gut microbial communities. These gut microorganisms are susceptible to alterations in the host system and can quickly evolve novel traits [15, 16], modifying host adaptability to GEC [30, 31]. Eventually, this mutually beneficial interaction between an earthworm and associated microbial communities may contribute directly to the holobionts’ adaptive strategies and initiate ecological and evolutionary feedback [30]. However, such ecological and evolutionary responses of earthworm holobionts to GEC remain largely ignored.

Here, we provide a conceptual framework to elaborate on the complex network of earthworm host–microbiota interactions and responses to GEC that jointly shape their ecology and evolution. Based on literature, we synthesize evidence of GEC impacts on earthworm holobionts and discuss evidence of ecological and evolutionary responses of earthworm holobionts to GEC. Lastly, we highlight the agro- and eco-system-level consequences of GEC-mediated shifts in earthworm holobiont functions.

### Evidence of global environmental change effects on earthworm holobionts

**Figure 1:**
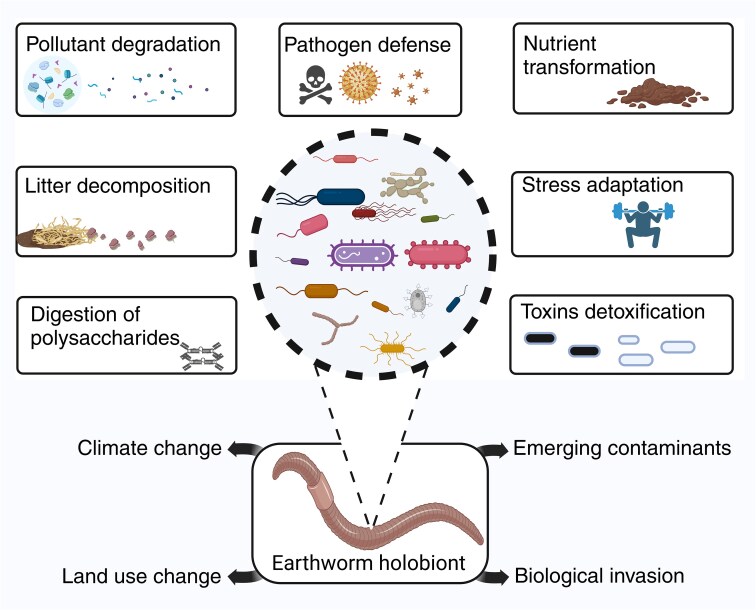
A conceptual framework elaborating on the interplay between the earthworm host and microbiota (i.e. earthworm holobiont) interaction and key global environmental change factors. Although the earthworm gut microbiota (microbes in thick black dash-circle) plays integral roles (functions in rectangular box) that positively influence the host and its environment, global environmental change (climate change, land use change, emerging contaminants, and biological invasion) strongly impacts the ecology and evolution of earthworm holobionts.

It is widely acknowledged that the global environment is rapidly changing and significantly affecting soil biodiversity and related biogeochemical processes [6, 32]. A large body of evidence suggests that such belowground alterations have shifted the dynamics of the complex soil fauna and microorganism interactions in many terrestrial ecosystems [21, 28, 33], considerably affecting nutrient availability and below- and aboveground productivity. Among the broad range of global change factors, climate change (elevated temperature or warming, CO_2_, and tropospheric ozone levels), nitrogen deposition, emerging contaminants (i.e. microplastics, heavy metals, agrochemicals, and antibiotics and other pharmaceutical residue), land use change (e.g. agricultural intensification and urbanization), and biological invasion significantly impact host–microbiome interactions. Despite growing awareness of GEC impacts on various earthworm ecological groups, knowledge of their influences on earthworm hosts and associated microbiota remains limited. Therefore, highlighting the possible effects and linkages of GEC to complex animal–microbe interactions is crucial for managing and utilizing belowground biodiversity (Fig. 1).

#### Climate change

Climate change is perhaps the most critical driver of Earth’s biodiversity loss [6, 34, 35] and can induce rapid host–microbiota responses [30], which are tightly linked with ecological processes and host fitness [15, 36]. Among the climate change variables, elevated soil temperature in response to climate warming exerts pronounced direct and indirect impacts on earthworm hosts and associated microbial communities [37]. While increasing soil temperature directly affects earthworm metabolic enzymes [38] and soil moisture through excessive evapotranspiration [39, 40], it indirectly affects substrate quality via increasing lignin concentration, disrupting earthworm host dietary preference [41].

Although litter quality is crucial for earthworm growth and has been shown to affect the earthworm microbiome [42], climate change–induced temperature increases critically alter litter quality [43], potentially affecting earthworm growth and functional characteristics [44]. Soil ecologists are interested in how earthworms adapt to such stressful conditions. For example, a shift in earthworm feeding preferences due to altered litter quality caused gut microbial communities to evolve traits suitable for cellulase enzymes, facilitating the digestion of complex cellulose in litter substrates and significantly reinforcing the earthworm host adaptability to changes in temperature and dietary resources [24]. Extreme temperature and drought have been reported to alter the stability of alpha diversity within gut microbiomes of individual hosts and generate beta diversity among microbiomes within the host population [45–47], impacting host fitness, microbial gene expression, and functional response to environmental stress [48]. Additionally, decreased litter quality induces competition between key earthworm host microbiota communities such as bacteria and fungi, modifying the microbial network complexity [45, 49]. Such modified microbial network complexity is supposed to buffer the host response to global change [45, 49]; however, competitive interactions within the microbiota in the gut system and ambient soil can potentially disorganize earthworm host recruitment of microbiotas, host–microbiome interactions, and functional dynamics. Although it remains untested in earthworm holobionts, dysbiosis and mass mortality have been reported in holobionts of the demosponge, *Rhopaloeides odorabile*, under elevated temperatures [50–52].

Elevated tropospheric ozone and CO_2_ concentrations significantly affect the diversity, composition, and productivity of soil faunas [53] and microbial communities [54, 55]. Elevated CO_2_ and tropospheric ozone have been found to modify litter quality, chemistry, and decomposition rate via increasing lignin concentration [48, 53], adversely affecting earthworm growth and host–microbiome interactions. For example, in a field study, elevated CO_2_ decreased the overall population composition of earthworms with a marked 25% reduction in anecic (litter feeding) earthworm biomass caused by a decreased litter substrate quality [56]. Most previous studies on elevated CO_2_ and ozone have primarily centered on litter quality–related impacts on earthworm hosts and soil physicochemicals (but see Chao *et al*. [45]), overlooking the possible consequences for host–microbiome interactions. However, a recent microcosm study showed that low-quality litter altered the alpha diversity of bacterial and fungal communities in the gut passage across all earthworm ecological groups [45]. As such, more studies (both field and pot experiments) are needed to deepen our holistic understanding of how earthworm host–microbiome interactions change in response to climate change and its ripple effects on the belowground biodiversity.

These climate change–induced disruptions suggest that earthworm host and associated microbiota response to climate change may be best explained at the holobiont level [57], offering an opportunity to study the reciprocal effects of ecological and evolutionary processes, i.e. how earthworms’ ecological responses to GEC may elicit the evolutionary response of their microbiota, ultimately affecting host evolution [36, 58, 59]. This may, in turn, selectively increase the recruitment of more beneficial gut microbiota, altering the abundance, diversity, and composition of gut microbiota. Such ecological and evolutionary feedbacks may underlie earthworm holobionts’ capacity to maintain functional stability under unfavorable environmental regimes. However, understanding whether such ecological and evolutionary feedbacks can be detected across all earthworm ecological groups, whether the magnitude of such responses is constant across all ecosystems, and whether the pattern of such eco-evolutionary responses remains constant regardless of environmental variation caused by climate change requires in-depth experimental exploration.

#### Emerging global contaminants

Large influxes of heavy metals [60, 61] and synthetic chemicals, including pesticides [62, 63], antibiotics or pharmaceutical residues [64, 65], and microplastics [66, 67] in soils have been found to exert detrimental effects on growth, abundance, and diversity of earthworm host-microbiota (see Table 1). For example, Xia *et al*. [4] recently found that elevated benzo[a]pyrene contamination in soils altered the adaptive strategies and ecological functions of earthworm gut viromes by disrupting the microbial metabolism and antiphage systems. Exposure to arsenic toxicity in soils changed the composition of gut microbial communities of *Metaphire sieboldi* earthworms [84]. While the mono-toxic effects of these soil contaminants have been a research focus over the years, the joint and synergistic impacts have been documented in recent decades [76, 85]. The gut microbiotas are noted for regulating host immune- and pathogen-related responses via metabolites and antimicrobial superoxide production [86, 87]. Therefore, contaminant-induced alterations of the abundance and diversity of earthworm gut microbiota may critically threaten host fitness, hormonal metabolism, and adaptation and responses to novel environmental cues [88]. As gut microbiota mediates hormonal stability and resilience of their host to chemical contaminants [89], the host buffers and stabilizes the gut microbiota communities by selectively recruiting more microbes with such beneficial impacts [60]. Such cooperative interactions have been demonstrated by exposing *Lumbricus terrestris* gut microbiota to cadmium contamination, which stimulated the abundance of heavy metal–resistant bacteria of the related genera (*Flavobacterium, Paenibacillus,* and *Pseudomonas*) that are vital for earthworm host adaptation and heavy metal remediation in soils [60].

**Table 1:** Evidence of emerging soil contaminant effects on earthworms and their gut microbial communities.

**Soil contaminant**	**Earthworm eco-type**	**Dominant gut microbiota**	**Exposure period (days)**	**Effects on host and associated microorganisms or ecosystems**	**Reference**
Pesticides	*Lumbricus terrestris*	Proteobacteria, Actinobacteria, Acidobacteria, Planctomyces, Verrucomicrobia, and Cyanobacteria	14	Pesticides decreased the total bacterial diversity in earthworms’ guts, even at the recommended application rate.	[68]
Herbicide (fomesafen)	*Pheretima guillelmi*	The phylae of Actinobacteria, Firmicutes, and Proteobacteria, as well as the genera of *Bacillus, Microvirga, Blastococcus, Nocardioides*, and *Gaiella*.	20	Exposure to fomesafen herbicide reduced the bacterial diversity energy resources and altered the gut community composition.	[69]
Microplastics (polyethylene)	*Eisenia fetida*	Proteobacteria (*Verminephrobacter* and *Bradyrhizobium*) and Firmicutes (*Bacillus*)	28	Polyethylene microplastic intake caused intestinal damage, altered behavior, and growth and weight loss.	[70]
Microplastics (polyethylene)	*E. fetida*	-	28	Exposure to polyethylene microplastics in the soil damaged DNA and caused transgenerational effects on earthworm reproduction of parents and offspring.	[71]
Arsenic	*Metaphire vulgaris*	*Rhodoplanes* and *Flavobacterium*	28	Arsenic exposure altered the gut bacterial community structure of the earthworm.	[72]
Microplastics	*M. guillelmi*	Actinobacteria, Planctomycetes, Firmicutes, and Chloroflexi		High-density polyethylene and polypropylene significantly altered the relative abundance of predominant phyla Actinobacteria, Planctomycetes, Firmicutes, and Chloroflexi.	[73]
Metal nanoparticles	*E. fetida*	*Verrucomicrobia*, *Acidobacteria Patescibacteria,* and *Proteobacteria,*	28	Metal nanoparticle toxicity negatively affected the relative abundance of *Verrucomicrobia*, *Acidobacteria,* and *Patescibacteria.*	[74]
Microplastics	*Eudrilus euganiae*	*Demequina*, *Nakmurella*, *Defluviicoccus*, *Azospria*, Clostridium, and *Demequina*	19	Earthworms exposed to polyethylene showed significant dysregulated enzyme activities that decreased the relative abundance of *Demequina* but increased that of *Nakmurella*, *Defluviicoccus Azospria*, and Clostridium.	[75]
Di(2-ethylhexyl) phthalate (DEHP)	*E. fetida* and *M. guillelmi*	Actinobacteriota, Firmicutes, and Proteobacteria	21	DEHP treatment decreased the dominant microbiota at the phylum level but increased the relative abundance of *Streptomyces, Thermobispora,* and *Gordonia*.	[76]
Diisononyl phthalate	*E. fetida*	Actinobacteria, Firmicutes, and Proteobacteria	28	Exposure to diisononyl phthalate significantly reduced the relative abundance of Chloroflexi and Patescibacteria at the phylum level.	[77]
Triclosan	*E. fetida*	Actinobacteria, Proteobacteria, and Bacteroidetes	7	Triclosan altered bacterial and eukaryotic community in the *E. fetida* intestine by increasing the relative abundance of Pseudomonas, Stenotrophomonas, and Achromobacter.	[78]
Heavy metal	*Eisenia andrei*	Proteobacteria and Bacteroidetes		Heavy metal contamination lowered the alpha diversity of gut microbiota.	[79]
Heavy metal	*E. fetida*	Burkholderiaceae, Enterobacteriaceae, and Microscillaceae	14	Exposure to heavy metals increased the abundance of Proteobacteria in the earthworm gut by 37.2%, but decreased that of Firmicutes by 2.01%.	[80]
Arsenic	*Metaphire* *sieboldi*	Actinobacteria, Firmicutes, and Proteobacteria	28	The abundance of Bacteriodetes and Streptomycetaceae increased with increasing arsenic exposure.	[81]
Microplastics	*E. fetida*	Actinobacteriota, Bacteroidota, Ascomycota, and Rozellomycota	28	Microplastic exposure altered the abundance of dominant microbial phyla in the earthworm gut.	[82]
Antibiotics	*M. guillelmi*	Actinobacteria	28	Polymyxin remarkably increased the abundance of Actinobacteria in the earthworm gut.	[83]

Indeed, earthworms ingest large quantities of soils from which specific microbial taxa are retained during the passage through the gut [11, 22]. Thus, the soil they ingest shapes the earthworm gut microbial communities [45]. In addition to acquiring these beneficial microbial communities, other soil contaminants, e.g. antibiotics and heavy metals, can accumulate and hamper the integrity of the earthworm gut microbiome [84, 90]. Similar impacts on other soil fauna have also been documented [91]. However, emerging evidence indicates that the earthworm gut generally exhibits a significantly lower number of antibiotic-resistance genes than the surrounding soil [92]. Similarly, arsenic contamination has also been shown to be associated with significantly lower arsenic biotransformation genes in the gut of the earthworm *M. vulgaris* than in ambient soils [72]. Microbial population disparities between the ambient soil and gut system, particularly the relatively low number of resistant genes in earthworm guts, could be a filtering mechanism or strategy for survival in an environment enriched with antibiotic resistance genes. Although such an adaptation is reported to decrease community productivity and diversity [28, 29, 93], it enhances and shapes the complex host–microbiota interdependency. Nevertheless, (i) whether such filtering mechanisms are initiated by the gut microbial communities or the host, (ii) whether similar filtering mechanisms occur in all earthworm ecological groups, and (iii) whether soil attributes also play a role in these patterns warrant urgent experimental validation to deeply understand the underlying mechanisms and their ecological relevance.

#### Land use change

The conversion of natural habitats through urbanization and agricultural intensification modulates the distribution and diversity of soil animals and microbial species [94–97]. Land use change critically impacts soil physicochemical characteristics, such as soil water content, structure, and permeability [98], potentially disrupting earthworm functional activities. Recent evidence suggests that agriculture-induced disturbances decreased earthworm species composition, richness, and diversity [99, 100] and increased the soil microbial network complexity and stability of soil fungal and bacterial communities [101]. Consequently, more complex microbial networks can lead to possible tradeoffs within gut microbial communities [45, 49, 101], shifting the dynamics of host–microbiome interactions.

**Figure 2:**
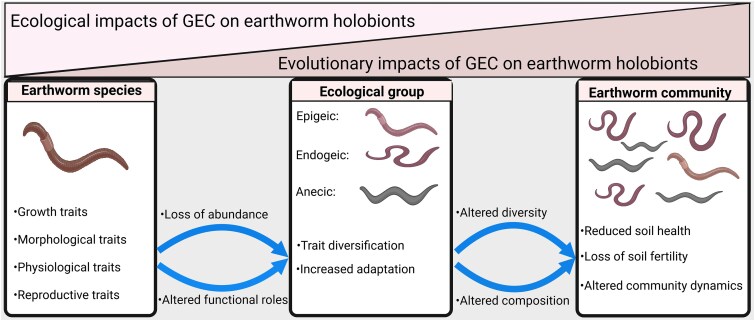
GEC impacts on earthworm holobionts. The ecological impacts showed stronger at the earthworm species level, i.e. on growth, morphological, physiological, and reproductive (traits in the first box). Contrary to the observed pattern of microbiome response to environmental change (adapted and modified from [16]), evolutionary impacts on earthworm holobionts remain unnoticeable at the species level but on the ecological groups. A loss of abundance and altered functional roles at the species level may lead to increased adaptation and diversification of traits across various earthworm ecological groups (traits in the middle box). However, adaptation and diversification of traits potentially lead to increased evolutionary consequences on various earthworm ecological groups, thus altering species diversity. Such impacts, i.e. loss of species diversity and thus change of composition across the overall ecological groups, ultimately affect the earthworm community, ecosystem functioning, and dynamics (last box).

Under land use–mediated disturbances of the soil environment, earthworm hosts may choose to migrate or adapt to avoid habitat modification impacts but may lag behind the rapidly changing environment [102]. Ultimately, the inherent rapid evolution of microbial communities associated with animal hosts may represent a vital alternative coping and adaptive mechanism to enhance resistance to such habitat perturbation [102]. However, the legacy effects of land use and agricultural intensification have frequently been shown to impact the composition and diversity of soil fungal and bacterial communities [101, 103]. This microbial assembly closely relates to earthworm gut microbial communities [45]. Given that the gut microbiome functioning depends on the individual microbial species and the interactive effect of the soil environment and microbial community structure [104], land use–induced alterations of the biotic component of the soil ecosystem can have strong implications on earthworm host–microbiota selection and ecological stability. While such alteration may impair the growth and fitness of individual earthworm species, it may exert stronger evolutionary consequences on the community due to natural habitat modification and a likely decoupling of beneficial host–microbiota associations (Fig. 2).

Land use intensity affects above- and belowground biodiversity [95, 105], especially of free-living soil microorganisms; however, microbial communities that are associated with other eukaryotes are conspicuously missing from such analysis (see Figs 1 and 2 of Gossner *et al*. [106] and Le Provost [105], respectively). While studies that specifically examine land use–related impacts on soil bacterial and fungi communities exist in literature [107–109], information on their counterparts living temporarily or permanently as pathogens or symbiotic partners remains scarce. As the free-living soil microorganisms and host–microbiota communities may differ in their responses to land use–mediated alterations as indicated elsewhere [110], such disparities can obscure the ecological realism of the impact of GEC, thus leaving most previously reported findings inconclusive. Although very few studies have addressed such impacts on earthworm communities, the focus has been on earthworm species without paying much attention to their associated microbial communities. Given the enormous contributions of earthworm holobionts to agricultural productivity and ecosystem stability, how land use change—urbanization, habitat fragmentation, and biotic homogenization—may influence earthworms and their associated microbiota communities and multifunctionality requires further exploration.

#### Biological invasion

Biological invasion is among the strongest drivers of GEC, influencing all aspects of below- and above-ground biodiversity [111–113]. Introducing non-native earthworm species into local communities profoundly impacts indigenous earthworm populations and earthworm–host microbiome interactions through competitive exclusion, habitat homogenization, altered soil properties, and a shift in soil microbial processes [113–115]. Generally, competition for limited available dietary resources and habitat space has been the underlying mechanism driving adverse effects of biological invasion on indigenous ecosystems [116, 117]. In a competitive environment, the adaptive response of native earthworm communities to invasive earthworms may decrease [118, 119], shift local niche partitioning, and increase community similarity [120, 121], potentially leading to altered microbial activities, soil respiration, and competitive exclusion of indigenous earthworm species with weaker competitive ability [122]. In Puerto Rico, for example, it was found that increased resource utilization significantly increased the population growth of the invasive earthworm *Pontoscolex corethrurus* compared to the native earthworms *Estherella* sp. and *Onychochaeta borincana* [122].

From an invasive plant viewpoint, a meta-analysis on plant invasions found that non-native species significantly mediated the loss of microbial diversity, ultimately shifting the microbial community structure of the native ecosystem [123]. Similarly, another meta-analysis suggests that invasive earthworms were associated with decreased plant diversity [124]. Such invasion-mediated modifications of native ecosystems markedly disrupt the diversity and distribution of other soil fauna (e.g. millipedes) [116]. However, detailed analyses of the impacts of invasive earthworms on the indigenous earthworm community and their gut microbial communities remain limited.

Alien plant invasion fundamentally alters plant community structure [115, 125] and soil biotic and abiotic components of the invaded ecosystem via exudates and allelochemicals [126–129], impairing native earthworm host–microbiota stability and ecological functions [130]. As such, Lobe *et al*. [131] found that removing the invasive shrub *Ligustrum sinense* significantly reduced the abundance of European invasive earthworms *L. rubellus*, *Aporrectodea caliginosa*, and *Octolasion tyrtaeum* and facilitated a 4-fold recovery of native earthworms in the North American forest. The authors suggested that removing *L. sinense* may have decreased the competitive advantage (i.e. modified leaf litter layer and increased soil nutrients and soil pH) conferred on European invasive earthworms [131]. Modification by invasive plants has also been reported to stimulate the increased similarity of soil fungal pathogen communities, leading to biotic homogenization of soil communities [132]. Additionally, invasive plants influence the abundance and diversity of local soil communities via priority effects, i.e. effects associated with the timing of arrival at the invaded site [133–135], critically modulating microbiome recruitment and the host–microbiota interactions of native communities.

North America remains a crucial hotspot and well-studied region for invasive earthworms from Europe and Asia [136]. Although earthworms are acknowledged as key ecosystem engineers, invaders can potentially cause substantial impacts on native soil biodiversity, critically impacting aboveground productivity through extensive soil mixing and leading to nutrient losses [124, 132, 137, 138]. For example, non-native earthworms have heavily invaded hardwood forests, drastically disrupting the soil microbiome and the abundance of functional genes [139] and potentially affecting soil biotic and abiotic resources. Specifically, invasive earthworm effects on arthropod communities [137], leaf-litter fauna [140], and soil fauna [141] have raised significant concerns in the northern part of North America. This unprecedented dominance of native ecosystems by non-native earthworm species can potentially drive a forceful dispersal of native earthworm communities, possibly triggering a loss of keystone gut microbiota species and symbiotic partners.

An extensive homogenization through the mixing of the organic layer can have cascading effects on soil chemistry, soil faunas [141], and soil microbial communities [142], with a strong effect on host-associated microbial communities [124, 143]. For example, Price-Christenson *et al*. [142] found that earthworm invaders in the genus *Amyntas* introduced novel assemblages of bacteria and fungi that altered the native soil microbial communities of the Wisconsin forest in the USA. The pathogenic species among these introduced bacterial and fungal invaders can hamper the host and associated microbiota via dysbiosis—imbalance of microbial species and reduction in diversity in the gut. Moreover, the cast of an invasive earthworm, *P. corethrurus,* significantly decreased total soil microbial and bacterial biomass in a microcosm study [144]. Therefore, invasive earthworms may modify the soil environment, which may have broader ecological consequences for native earthworm host–microbiota interactions, soil health, and aboveground productivity.

Despite the potential impact of invasive earthworms on soil biodiversity, microbial communities, and soil fauna diversity, the effects of invasive earthworms on gut microbial communities of native earthworm assemblage remain a significant research gap in earthworm invasion studies. As plant invaders are known to influence the dynamics of ecosystems they invade via altered abundance and diversity of native plants and associated microbial communities [127, 145, 146], disruptions of earthworm hosts and their associated microbial communities may similarly have considerable ecological and evolutionary consequences in earthworm-invaded ecosystems. Indeed, there is a vast knowledge deficit on how invasive earthworms may directly and indirectly affect host–microbiota interactions of their native earthworm congeners. To deeply understand such direct and indirect effects of biological invasion on earthworm holobionts, many other pending questions may need answers through experimental explorations. For instance, (i) do the responses of native earthworms and their microbiome to invasive earthworms’ impacts differ among the various ecological groups? (ii) Do the responses of native earthworm communities depend on specific gut microbial taxa that enhance biotic resistance to earthworm invasions? (iii) Does increasing native earthworm diversity promote community resistance to future earthworm invasion, as Elton’s diversity−invasibility hypothesis predicts [147, 148], and (4) does earthworm invasion affect the coupling relationship of other vital host–microbial interactions such as plant-arbuscular mycorrhizal fungi (AMF) partnerships? (Table 2).

**Table 2:** Evidence of invasive earthworm effects on biotic and abiotic components of terrestrial ecosystems.

**Component of terrestrial ecosystems**	**Region/Specific ecosystem**	**Invasive earthworm species**	**Ecosystem impact**	**References**
Soil microbial communities	Subtropical forest	*Pontoscolex corethrurus*	Reduce total soil microbial biomass and bacterial biomass.	[144]
	Wisconsin forest, USA	*Amynthas* spp.	Introduce novel bacterial and fungal communities through their cast.	[142]
	Minnesota hardwood forest, USA	Multiple species	Alter soil microbiome structure and functional gene abundance.	[111]
	Temperate hardwood Forests	*L. terrestris, Aporrectodea turgida, A. rosea,* and *Dendrobaena octaedra*	Alter soil respiration and carbon cycling.	[149]
Soil faunas	North American forests	Multiple species	Reduce soil fauna abundance and diversity.	[137]
	Hardwood forest, USA	Multiple species	Decrease the abundance of soil-dwelling arthropods.	[150]
	Field study^a^	*P. corethrurus* and *Amynthas corticis*	Reduce species richness of nematode communities.	[151]
	Northeastern North American Forest	Multiple species	The abundance of invasive earthworms negatively correlated with the abundance of leaf-litter fauna.	[140]
	Brazilian Atlantic Forest	*P. corethrurus, A. corticis,* and *A. gracilis*	High soil bioturbation caused by invasive earthworms threatened the macroinvertebrates.	[114]
Soil physical and chemical attributes	Wisconsin forest	*Amynthas* spp.	Alter soil chemistry.	[142]
	Field study^a^	Multiple species	Alter soil abiotic properties such as soil pH and nutrient availability.	[152]
	Minnesota hardwood forest, USA	Multiple species	Increase the proliferation of denitrification genes, and alter nitrogen cycling.	[111]
	Field study^a^	*P. corethrurus* and *A. corticis*	Reduce the content of soil nitrates and nutrient availability.	[151]
Plant growth and biomass	Microcosm study^a^	*L. terrestris* and *Aporrectodea rosea*	Alter plant functional traits such as height and root length.	[153]
	Field study^a^	Multiple species	Change plant functional diversity.	[121]
	Northern hardwood Forest	*Lumbricus* spp.	Reduce plant species richness.	[154]
	Field study^a^	*Aporrectodea trapezoids*, *A. caliginosa*, and *Allolobohora* spp.	Increase the emergence of invasive plant seedlings and species richness.	[155]
	North American forest	Multiple species	Modify the dominance of fast-growing plant species and alter understory community traits and functional diversity.	[121]
	Northeastern North American forest	Multiple species	Mediate the reduction of native plant communities.	[125]
	Forest understory	Multiple species	Alter nutrient availability and plant nutrient uptake from soils.	[156]

a Controlled study.

### Evidence of ecological and evolutionary responses of earthworm holobionts to global environmental change

Responses of earthworms to GEC have been reported at both species and community levels with varying impacts on all ecological groups [157]. Because the various ecological groups of earthworms vary in functional traits and niche partitioning [158], responses to environmental change impacts may also differ accordingly. Moreover, an adaptive response to GEC at the host–microbiota level largely depends on environmental context, host species, and microbiota identity [2, 23]. Regardless of these differences, host-associated microbial communities play significant roles in the adaptation strategies of hosts to increase fitness and survival [157]. As a vital driver of co-evolutionary adaptations [159], the associated microbial communities and their earthworm host intimately drive overall ecosystem functioning and productivity dynamics. The need to know whether earthworms and associated microbiota may co-evolve novel traits and respond to such environmental perturbations underscores the need to advance knowledge on the transformational dynamics of roles of animal host–microbiota interactions in terrestrial ecosystems. We propose five ecological and evolutionary responses that may mediate performance and fitness consequences in the earthworm holobiont system.

#### Diversification of functional traits of earthworm holobionts

In 1881, Darwin (1809–82) recognized earthworms’ activities as indispensable for maintaining soil fertility through soil organic matter mineralization, nutrient availability, and plant growth [160]. However, these essential functional attributes of earthworms have significantly been compromised since the advent of the Green and Industrial Revolution through increasing human-driven inputs of both organic and inorganic compounds, e.g. pesticides, fertilizers, and microplastics [75, 161, 162]. As a survival strategy in earthworms, the gut microbiota has evolved novel enzymes and traits capable of degrading organic and inorganic compounds [163, 164]. For instance, Gram-positive bacteria of the phylum Actinobacteria and Firmicutes isolated from the gut of the earthworm *L. terrestris* significantly degraded microplastic particles in the soil by 60% in a macrocosm study [164]. Accordingly, after evaluating the global microbiome for plastic-degrading enzymes, Zrimec *et al*. [163] found 18 119 soil enzymes that can degrade nine different polymer types, providing an evolutionary adaptation capacity to these microorganisms. This gut-mediated adaptive strategy provides evidence for intense selective pressures on these microorganisms in soils with high levels of microplastic polymers. This suggests microbiota communities may be pivotal in earthworm host niche differentiation over ecological and evolutionary timescale [165].

Furthermore, earthworm hosts may reinforce such adaptive or coping mechanisms by recruiting more gut microbiota with similar organic and inorganic degrading traits to buffer the newly acquired niche (i.e. compounds degrading abilities), shaping their evolution. One primary gut microbiota–mediated coping mechanism is the secretion of antioxidant enzymes capable of degrading organic compounds such as microplastics [157], emphasizing the potential of earthworms as bioremediation agents in terrestrial soils [157, 166, 167]. Recent analysis of soil microplastic effects on the earthworm *E. fetida* revealed that specific microplastic types can activate these antioxidant defense mechanisms [168]. However, such ecological diversification (i.e. the evolution of divergent ecological traits within a lineage) of original traits may impose two major ecosystem challenges. First, once achieved, ecological diversification remains irreversible as it originates from more generalized ancestors to specialized progenies or descendants [169]. Second, an adaptation is shown to decrease productivity at the ecosystem level due to a loss of dominant keystone microbiota community or ecological tradeoffs [28, 29, 93]. While the various earthworm hosts fundamentally differ in their ecological roles and niche differentiation, increasing adaptation within a population can lead to intrahost evolution (also known as within-host evolution) and the emergence of a hybridized population [170] with strong consequences for untimely extinction of the core microbiota, compromised host defense chemistry, and homogenized host–microbiota interactions.

#### Microbially acquired novel resistance genes via horizontal gene transfer and transgenerational plasticity

Contamination of terrestrial soils with the “so-called” emerging contaminants, including antibiotics, heavy metals, and microplastics [171, 172], has substantially surged owing to their increasing application in agrosystems worldwide [173]. Although these chemicals are increasingly applied or used for their beneficial roles in agricultural production, their soil residues induce collateral effects on nontarget beneficial microbiota and the host systems. For instance, previous studies have reported varying degrees of direct effects of antibiotics on earthworm gut microbiota diversity and physiology through impaired hormonal metabolism, growth, and reproduction [91, 174, 175]. Thus, antibiotics significantly impact the stability of the host–microbiota interactions and collective ecosystem services [176]. However, with their rapid adaptability and swift evolutionary response to environmental stress, some microbial communities may quickly adapt to antibiotic exposure by acquiring antibiotic-resistance genes. These antibiotic- and metal-resistance genes are acquired through horizontal gene transfer and transgenerational transfer from parents to progenies.

##### Horizontal gene transfer

Horizontal gene transfer is the transfer or exchange of genetic materials or information between organisms [177, 178]. Antibiotic and metal resistance genes can traverse membranes and optimize an organism’s capacity to resist future exposure to contaminants and acquire greater selective pressure in their environment. In natural ecosystems, selective pressure has been quantified by measuring the abundance of the *intl* gene (i.e. class 1 integron-intergrase gene), which has widely been used as a proxy for selective pressure associated with environmental contaminants [179, 180]. The majority of recent analyses of contaminant-related effects on earthworms and other soil fauna have reported the presence of *intl* in earthworms [173, 181–183], suggesting that both hosts and gut microbiota species have evolved adaptive mechanisms to resist the toxicity effects of emerging contaminants.

##### Transgenerational plasticity

**Figure 3:**
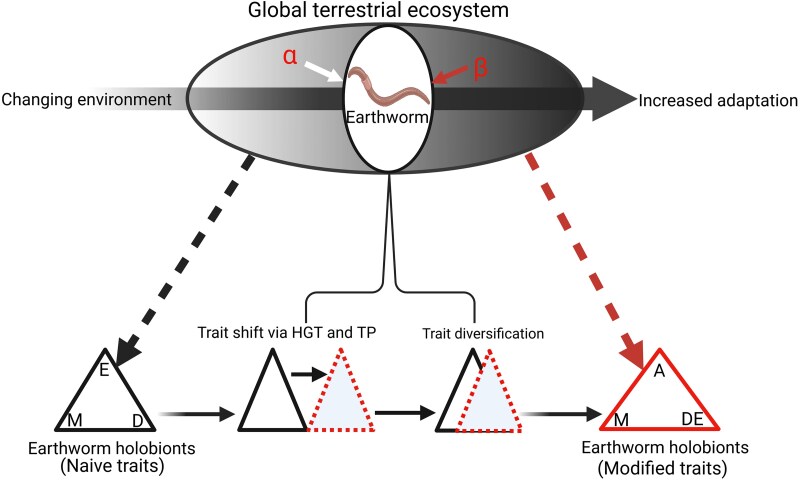
Pathways of earthworm holobiont responses to global environmental change. In the terrestrial ecosystem, earthworm hosts recruit microbiota from the ambient soils. These microbiotas may be located in the naive zone (α, i.e. microbiotas less adapted to environmental change factors in the ecosystem) or experience zone (ꞵ, i.e. microbiotas highly adapted to environmental change factors). Thus, amid environmental change, earthworm holobionts with naive traits survive by the environment (E), microbiome (M), and dietary sources (D) (triangle connected by thick black-dash arrow). However, under the changing environment, earthworms acquire novel traits through horizontal gene transfer (HGT) and transgenerational plasticity (TP) (black- and red-dash triangles in contact), leading to trait diversification (merged red-dash and black triangles). With trait diversification, earthworm holobionts acquire modified traits or increased adaptation but will likely lose symbiotic partners due to trait and dietary shifts. Earthworm holobionts with modified traits or increased adaptation may survive in the changing environment (red triangle connected by the thick red-dash arrow) by adaptive traits or strategies (A), microbiome, dietary sources, and the environment.

Transgenerational plasticity is the modification of offspring phenotypes that increases fitness and survival in response to environmental conditions experienced by parental generations [184–187]. In a cross-generational metal resistance analysis, it was observed that the second-generation offspring (F2) of the earthworm *E. fetida* were less affected by oxidative stress than the first-generation (F1) parents when exposed to gradients of heavy metal–contaminated soils [181]. Thus, Cd bioaccumulation and detoxification genes of metallothionein significantly increased by 150% and 296% from the F2 to the F1 generation, suggesting higher metal resistance in F2 [181]. Similarly, in an earlier study, the F1, F2, and F3 generations of the earthworm *L. rubellus* derived from arsenic-contaminated fields were all highly tolerant when exposed to 2000 mg Kg^−1^ of arsenic toxicity [188]. Likewise, the soil detritivore collembolan *Folsomia candida* in soils contaminated with heavy metals was found to reproduce 5 times more than that in neutral soils and 20 times more than that in calcareous soils in a multigenerational study spanning five generations [189]. However, this adaptive transgenerational response in *F. candida* was accompanied by a tradeoff between reproduction and detoxification of accumulated metals in the detritivore body. More studies are urgently needed to highlight the role of associated microbiotas in such ecological and evolutionary strategies mediating host adaptation to contaminant toxicity in terrestrial ecosystems (Fig. 3).

#### Diversification of earthworm host-symbiotic partners

In holobionts, the host and associated microbiota are considered an ecological unit with a common genome called hologenome [3, 190]. Thus, partners complement one another’s genetic system to modify their collective adaptation and evolution. Indeed, this interaction can develop to the point that each partner survives at the expense of the other. However, a change in dietary resources and habitat conditions can significantly spur ecological diversification (i.e. the evolution of divergent ecological traits within a lineage [169]) of a partner to optimize adaptation to or escape impending stressors. When a partner’s newly evolved traits do not benefit the overall host–microbiota system, it may likely lead to the expulsion of a species from the symbiotic partnership. Analogous to an ecological diversification event is host switching (changing host) that mainly occurs between bacterial symbionts—*Verminephrobacter* and *Flexibacter*-like bacteria—that inhabit the excretory organ (nephridial) of lumbricid earthworms [191–193]. For example, in an earlier phylogenetic analysis to investigate the evolution of a tripartite symbiosis in 18 lumbricid earthworm species, distinct evolutionary histories were found for the two key nephridial symbionts *Verminephrobacter* and *Flexibacter*-like bacteria [191]. Thus, while the symbiont phylogenies of *Verminephrobacter* in the lumbricid earthworm nephridial signaled a long-term co-evolution with the host, that of *Flexibacter*-like symbionts suggested a switch to a new host that promoted their adaptation [191]. Therefore, diversification of symbiotic partners in earthworm holobiont in response to GEC may harm the holobiont system, consequently affecting host fitness, especially reproduction, and broader ecosystem functions.

Ongoing GEC disruptions within the plant host–microbiota environment have led to the formulation of various hypotheses, including adapt or disperse [194], adapt or migrate [195], and partner or perish [196], to explain ecological consequences of mutualism or host–microbiome interactions under challenging times [197, 198]. However, hypotheses concerning animal–microbial interactions, especially the earthworm host and their associated microbial communities, remain largely ignored. Under stressful conditions, the earthworm host and its associated microbial communities evolve varying responses to increase survival, leading to their coevolution [199]. Such coevolution may increase the selective pressure on their hologenome, up-regulating their chances of resistance against similar future environmental pressures. Despite the usual host sanctions and punishment for underperformed partners in mutually symbiotic systems [200], earthworm microbiota may likely undergo partner or perish and adapt or disperse scenarios without sanctions. The reason is that earthworms usually do not involve all their gut microbiota in all stressful conditions but tend to recruit species-specific microbiota capable of contributing to the abatement of the stress [1]. For instance, the microbial taxa recruited to facilitate the resistance against hot temperatures, drought, and chemical toxicity may differ. This suggests that coevolution in earthworm holobionts may be context-dependent and microbiota taxa-specific, modifying their fitness, survival, and adaptation of holobionts to variable environmental regimes. These valuable insights require further experimental exploration to understand further the interplay between hosts and the vast array of microbial consortia in their holobiont system.

#### Biological invasion-induced shift in microbial assembly

Both non-native plants and animals affect the belowground biodiversity of native communities, especially the soil biota, with cascading impacts on their beneficial roles [201–203]. From a plant viewpoint, invasive plants alter the soil biota communities by modifying litter quality and rhizosphere inputs [203]. For instance, a meta-analysis showed that the modification of litter quality by invasive plants increased the abundance of soil detritivores and microbivores by 119% and 89%, respectively, while fungal biomass increased by 36% and bacterial biomass decreased by 12% in the rhizosphere [203]. Also, the invasive species-mediated decrease in beta diversity and increase in alpha diversity of soil microbiome have also been documented [202]. Thus, invasive plants can indirectly influence earthworm holobionts by altering host abundance and diversity. Among key mechanisms underlying such invasive plant-induced effects on soil biota include nutrient availability and allelochemicals in the form of root exudates and litter leachates, as indicated by the famous novel weapon hypothesis [204, 205]. As earthworms depend primarily on litter as a dietary source, manipulating litter quality via allelochemical exudates can significantly affect host growth, reproduction, and functions.

Plants produce abundant polyphenols, suggesting that earthworms likely ingest high quantities of these compounds through litter feeding [206]. For this reason, earthworms show a preference for litter substrates of low polyphenols as they cause protein precipitation [207]. Nevertheless, it is reported that earthworms can alleviate the harmful effects of plant polyphenol-rich compounds in litter materials via key adaptation mechanisms [157, 207]. Liekebe *et al*. [207] indicated that earthworms strategically overcome such allelochemical impacts with a compound called drilodefensin (i.e. a unique surface-active metabolite in their gut), which can counteract the adverse effects of polyphenols on earthworm gut enzymes. Despite numerous reports of allelochemical impacts on plant–plant [127] and plant–soil microbe interactions [208], responses of earthworm hosts and associated microbial communities to allelochemical compounds remain unclear.

From an animal viewpoint, invasive animals can profoundly impact soil fauna and associated microbial communities through biochemical metabolites [209]. For instance, the metabolites in earthworm mucus reduced reproduction, increased mortality, and altered bacterial feeding preference of soil-dwelling nematodes [209]. As novel biochemical weapons, allelochemical exudates in soils likely impose selection pressure on the earthworm microbiome community to increase host adaptation and resistance due to the rapid evolution of microorganisms [16]. However, how other invasive animals can impact earthworms and their associated microbial communities and their subsequent responses remains unknown. Given the extent of damage that biological invasion inflicts on belowground biodiversity, the ecological and evolutionary responses of earthworm holobionts to biological invasion must be fully comprehended.

#### Environmental change–induced shift in dietary preference

Climate change–induced alteration in the dietary preferences of soil animals is among the factors driving belowground biodiversity loss [210, 211]. As plants adapt metabolically to cope with, for example, ongoing elevated warming, drought, and CO_2_, there may be significant changes in plant secondary metabolites [32, 212]. As such, a shift in plant metabolites may strongly influence litter quality and soil inputs [213]. Such changes may profoundly alter earthworms’ dietary preferences by selecting metabolic traits and mechanisms and recruiting gut microbial communities with strong adaptations to enhance the digestion of these new litter substrates. Thus, climate change effects on plant litter quality may indirectly drive an ecological and evolutionary response of earthworms and their microbial communities. This scenario has been recently demonstrated by Yang *et al*. [24], reporting that the earthworms *E. nordenskioldi* and *D. ghilarovi* strategically recruited cellulose-degrading gut bacterial communities (e.g. Actinobacteria, Planctomycetes, and Firmicutes) to increase host adaptability to increased temperature and cellulose digestion. As indicated in the previous section, the evolution of a unique surface-active metabolite in the gut (i.e. drilodefensin) of earthworms buffers their capacity to cope with dietary challenges of polyphenol-rich compounds in plant litter [207].

An overwhelming body of evidence has indicated that warming causes range shifts or expansion of plants and animals [33, 136, 194, 214], with critical implications for biotic interaction between host and associated microbial communities [113, 215]. Such range expansion is mainly accompanied by radical trait changes and evolutionary shifts favoring the ability to thrive, establish, and competitively dominate in native ecosystems [216, 217]. A notable occurrence of warming-induced shifts is the invasion of European earthworms in the North American ecosystem [113, 139–141]. Such a range shift could pose two major bidirectional impacts at both the ecological and evolutionary levels. First, range shift may lead to biotic homogenization of the local communities and functional composition of keystone species, compelling hosts to re-assemble novel microbial taxa with unique traits to modify host–microbiota adaptation. Second, the native communities may be required to evolve unique traits to adapt and co-exist with invaders or migrate, homogenizing the structure of invaded ecosystems. Although climate change effects have been extensively studied across many species [218, 219], such collateral impacts on earthworm holobionts remain a significant research gap in our current understanding.

### Agro- and eco-system consequences of loss of earthworm host–microbiota interactions

Earthworms have a long-standing history of improving soil health and ecosystem productivity through litter feeding, soil-burrowing activities, improving soil infiltration, and restructuring soil biological properties [7, 220]. During herbivore attacks, earthworms modify plant resistance by stimulating plant nutrient uptake that increases biomass accumulation and mediates the expression of phytohormonal pathways [221]. Evidence has emerged that certain key members of earthworm microbiota regulate fungal pathogens and nematodes in agro- and eco-systems [222]. Despite earthworms’ numerous vital cooperative roles with their associated microorganisms, increasing adaptation and ecological diversification in response to the rapidly changing environment can substantially affect their ecosystem functions and beneficial effects in agrosystems [223]. As indicated earlier, increasing adaptation and trait diversification to ensure fitness and survival under environmental change decreases organisms’ productivity due to significant resource allocation for optimum performance. Given the importance of earthworms and associated microbial communities, GEC-induced modifications in their functional traits and ecological niches can hamper their agro- and eco-system relevance.

GEC-mediated loss of functional diversity of the various ecological groups may significantly impact key biogeochemical processes such as litter decomposition, which has strong consequences for ecosystem nutrient availability [129, 224]. A recent meta-analysis showed that earthworm functional group diversity modulates litter and organic carbon decomposition in soils [225]. In agrosystems, soil nutrient limitation indirectly represents additional inputs of synthetic chemical nutrients to ensure maximum productivity at the expense of numerous unintended adverse effects on soil biodiversity. Moreover, the earthworm gut microbiome plays a pivotal role in the biogeochemical cyclings of microplastics, antibiotics, and toxic metals—important aspects of soil remediation [167, 226]. This implies that a change in earthworm abundance, diversity, and functional traits may represent a significant buildup of toxic compounds, antibiotics, and disease proliferation in agrosystems. Indeed, the buildup of antibiotics and pathogens may indirectly threaten food security and global health. Therefore, instead of allowing this trait diversification of earthworm holobionts to impose unprecedented challenges in agrosystems, such traits could be harnessed and engineered as a biocontrol measure against disease pathogens and remediation of toxic metals from agricultural soils.

### Future perspectives and outstanding questions

As indicated earlier, global warming and elevated CO_2_ critically influence plant secondary metabolites, impacting litter quality and modifying earthworm dietary preferences. Although litter quality has been found to modulate the earthworm microbiome [42, 45], very few studies have examined how GEC-induced litter modification may impact earthworm host–microbiota functioning and ecosystem nutrient dynamics. Experimental designs should consider the interactive effects of global change factors as these factors do not operate in isolation. For example, recent studies have considered land use and climate effects on earthworm and soil microbial communities [17, 227] and responses of soil biodiversity to climate and land use change [228]. The earthworm ecological groups differ in many aspects, including their morphology, physiology, feeding activity, and associated microbial communities, creating differing niches. However, whether such niche differentiation among earthworm ecological groups may influence the response of their gut microbiota and the overall host–microbiota fitness and activities needs experimental clarification.

Unlike invasive plant impacts on native ecosystems that have received extensive recognition, the range expansion of invasive earthworms and their effects on native earthworm communities, especially in North American ecosystems, remain limited. Moreover, the extent to which invasive earthworms may affect the key gut microbiota communities of native earthworms and the mechanisms driving such impacts are nearly unknown. Given the joint ecological roles of earthworm host–microbiota in litter decomposition and ecosystem nutrient availability and dynamics, extensive studies are needed to explore how invasive earthworms affect native earthworms and their associated microbial communities. In particular, studying the interaction of climate change factors and invasive earthworms’ effects on native earthworms and other soil macrofauna may be very useful. Such knowledge will broaden our general understanding of the effects of biological invasion on both local floras and faunas. Finally, earthworm gut microbiotas have evolved novel traits that enhance their ability to degrade soil contaminants. Such trait modification or diversification is likely to create tradeoffs; whether such tradeoffs may pose fitness consequences on earthworms’ ecological roles needs clarification.

## Conclusions

Earthworm holobionts represent a complex interdependent system, performing many biogeochemical functions and shaping the ecology and evolution of hosts and their symbiotic partners under GEC. The multifaceted impacts of GEC substantially influence the physiology, behavior, and functional dynamics of earthworm holobionts, underscoring the need to study the interplay between the ecological and evolutionary processes involved in their responses. Such knowledge may be crucial for predicting and quantifying species- and community-level impacts of environmental change on soil detritivores. Indeed, earthworm holobionts remain understudied, although such understanding can potentially be linked to unraveling the responses of other vital soil detritivores and their microbial communities to GEC. Therefore, closing the gap between the ecological and evolutionary responses of earthworm holobionts may serve as a starting point for a global reconsideration of holobionts in the ongoing environmental changes.

## Data Availability

Data sharing is not applicable to this article as no datasets were generated or analyzed during the current study.
